# Prevalence and factors associated with suicidal ideation and attempts among war-affected internally displaced people in northwest Ethiopia, 2022

**DOI:** 10.1192/bjo.2024.71

**Published:** 2024-08-01

**Authors:** Gebresilassie Tadesse, Fanuel Gashaw, Tadele Amare Zeleke, Setegn Fentahun, Sewbesew Yitayih

**Affiliations:** Department of Psychiatry, School of Medicine, College of Medicine and Health Sciences, University of Gondar, Ethiopia

**Keywords:** Suicide, suicidal ideation, suicidal attempt, internally displaced people, Ethiopia

## Abstract

**Background:**

People who are forced to leave home often experience emotional suffering and may be disproportionately subjected to risk factors for suicide. Although it is a grave concern for the global public health community, it has not been understood in Ethiopia.

**Aims:**

This study aims to assess the prevalence and factors associated with suicidal ideation and attempts among war-affected internally displaced people in northwest Ethiopia, 2022.

**Method:**

From 23 May to 22 June 2022, a cross-sectional study design was conducted, and a sample of 765 participants was selected through simple random sampling. A structured interview was employed to collect data. Suicidal ideation and attempts were assessed using the Composite International Diagnostic Interview.

**Results:**

Out of 751 interviewed participants with a response rate of 98.2%, the magnitude of suicidal ideation and attempt was 22.4% (95% CI: 19.5%, 25.4%) and 6.7% (95% CI: 5.1%, 8.7%), respectively. People of female gender, having depression, family with a history of mental illness, and poor social support were significantly associated with both suicidal ideation and attempts. Furthermore, post-traumatic stress symptoms and the death of a family member were significantly associated with suicide ideation and attempt, respectively.

**Conclusion:**

At least one in five of the displaced people in this population had experienced suicide ideation, and one in fifteen had attempted suicide. Therefore, strengthening early detection and intervention for individuals is recommended, especially for females with depression, post-traumatic stress symptoms, family with a history of mental illness, poor social support and the death of family members.

Suicidal ideation refers to thoughts about taking one's own life, either actively (with a plan) or passively (with only a wish to die and no plan). On the other hand, a suicidal attempt is an intentional act of harming oneself in an attempt to end one's life, which could not be fatal because of a variety of factors, including other people's intervention.^1^ Suicide has attracted more attention and research in public awareness campaigns than before, and has been a key area of concern for the global public health community.^2^ Every death by suicide is a personal tragedy that affects a number of individuals subsequently, including friends, family and communities.^3^ Both suicidal ideation and attempts are highly predictive of death by suicide, and can lead to injury, hospitalisation and billions of dollars in financial burdens on society.^4^

Evidence shows that suicidal ideation and attempts are a major global concern, especially among internally displaced people and refugees.^5^ Internally displaced people (IDP) are frequently perceived as unwelcome minorities, which exposes them to greater discrimination and social exclusion.^6^ In response to the loss of their home, security, possessions and loved ones, they may experience varying degrees of stress and hopelessness and feel upset about the situation or the individuals who evicted them from their homes.^7^ Low- and middle-income countries (LMICs) account for 80% of suicides globally, but only 10% of all published literature on suicide is conducted in LMICs.^8^ Evidence, however, points to potential differences between the character and progression of suicidality between LMICs and high-income countries.

Notably, studies conducted in financially secure nations frequently discover that the rate of suicidal thought is higher than that of actual suicidal behaviour.^9^ These results could point to distinct trends in the transition from ideation to attempt among populations in LMICs, despite the possibility that stigma and taboos around suicide make people less likely to disclose their thoughts before acting on suicide.^10^ Furthermore, there may be differences in the type and degree to which particular factors account for suicide in high-income and low- and middle-income nations. For instance, meta-analytic data suggest a weaker relationship between psychopathology and suicidality in LMICs compared with high-income countries. It emphasises the significance of comprehending how interpersonal and contextual factors like poverty, social support, and family and community violence affect people's risk of suicide in resource-constrained settings.^11^

In order to increase the generalizability and efficacy of suicide prevention initiatives, more study is urgently needed on the magnitude and determinants of suicide among representative at-risk populations in these settings.^12^ Displaced people living in temporary camps in regions with limited resources might be particularly vulnerable to suicide because of the particular challenges they face in their surroundings, including high rates of community and family violence, alterations to social support networks, crowded housing, an inadequate supply of basic needs, and exclusion from jobs and opportunities for education.^13^

In accordance with a report from the World Health Organization (WHO), suicide takes a single life every 40 s,^8^ and there are 800 000 suicide deaths globally each year.^14^ There have been variable reports on suicidal ideation and attempts among IDP and refugees in Colombia (19.8%, 9.1%),^15^ Mississippi (20%, 3%)^16^ and South Korea (28.3%, 17.3%),^17^ respectively. A thorough assessment and meta-analysis of 77 studies was carried out, with a particular emphasis on five different categories of displaced people of all ages. These subgroups include IDP, refugees residing in camps, refugees granted permanent asylum, and mixed samples of refugees and asylum seekers. Suicidal ideation and attempt rates varied from 0.17 to 70.6% and 0.14 to 15.4%, respectively, in community samples across all demographics.

Suicidal ideation and attempts were common among refugees residing in camps, with rates ranging from 1.2 to 32.3% and 0.18 to 7.3%, respectively. A study from African countries shows that the burden of suicidal ideation among IDP ranged from 0.9 to 33.8%,^18,19^ whereas a study carried out in Tanzania on 460 Burundian refugees revealed that the magnitude of suicidal ideation and attempt was 37.4%, 5.2% among females, and 29.6%, 1.7% among males, respectively.^10^ According to a systematic review conducted in Ethiopia among homeless individuals, the pooled current prevalence of suicide ideation and attempt was 17.83 and 9.16%, respectively, while the lifetime prevalence was 41.6 and 28.8%.^20^ A more complete picture of suicide has been made possible through developments in our understanding of the variables that influence suicide risk. A higher risk of suicide was predicted to be associated with substance abuse, recent family loss, mental illness and a low sense of social support. According to a recent meta-analysis study, 26.4% of IDP suffer from depression.^21^ Individuals with depression usually experience warning signs including hopelessness, loss of pleasure, acting out behaviours and running away, thoughts of wanting to die, increased physical complaints and effort towards plans to die by suicide. The risk of suicide in people with untreated depressive disorder is approximately 20%,^22^ and in people with major depressive disorder, the risk of suicide is approximately 20 times that of the general population.

Overall, displaced people are exposed to different kinds of trauma, violence and injuries, making them more vulnerable to psychological disorders, particularly post-traumatic stress symptoms.^23^ According to a systematic review carried out in 40 nations, 30.6% of war-affected IDP have experienced post-traumatic stress symptoms.^24^ Disturbing traumatic memories, frustration and a lack of impulse control are among the signs of post-traumatic stress disorder. Suicidal thoughts may occur in someone who experiences flashbacks and reminders of their fear and terror because these feel impossible to control.^25^

Substance use was one of the main predicting factors for individuals exposed to suicidal behaviours. Substance users usually become hopeless and have feelings of unhappiness, regression, loneliness and self-criticism.^26^ According to Danielle Horyniak's review findings,^27^ about 17–36% of displaced people were substance users, of alcohol especially. A common reason why people use drugs and alcohol is to either conceal memories of past experiences or to dull uncomfortable emotions. This frequently results in unhealthy avoidance and more profound depression. A massive hurdle that interferes with hope is formed through a combination of broken relationships, health problems, job loss and financial difficulties. Self-criticism frequently results in depressed feelings and the impression that no one seems to attest to the person's suffering. Sometimes the pain of loneliness is so overwhelming that the only means of getting out appears to be suicide.^26^

Nevertheless, research on whether displaced people genuinely have a higher risk of suicide than host communities has been inconsistent and appears to depend on the particular situation. The absence of studies concerning displaced individuals is a significant gap in the current understanding of suicidality among IDP, even though the interpretability and comparability of the various incidence rates of suicidality rely on contextual factors. Suicide may not usually be reported, and those who are in danger of suicide may be reluctant to seek treatment because it is widely stigmatised or even banned in several nations.^28^ Lastly, there is a scarcity of research into possible associations between contributing factors and suicide among internally displaced individuals.

Therefore, the current study sought to investigate the prevalence of suicide ideation and attempts among IDP residing in camps in northwest Ethiopia in an effort to help close these gaps. Although determining the prevalence of suicidal ideation and attempts is essential to exploring the scope of the issue in this at-risk and understudied population, identifying and altering locally pertinent risks and protective factors is important for creating and putting prevention efforts into practice. Thus, this study additionally sought important variables related to suicide.

## Methods and materials

### Study design and period

This study was carried out from 23 May to 22 June 2022, through a cross-sectional study design.

### Study setting

Ethiopia has been dealing with the crisis of internal displacement for decades and is the host of a large number (more than 2.2 million) of displaced people in the Horn of Africa due to inter-communal violence in 2018 and 2019, of whom 1.4 million were displaced in 2019. The confrontation between the federal government and the Tigray People Liberation Front (TPLF) has intensified, and millions of people in the Tigray, Amhara and Afar regions have been displaced. Many children, adolescents, women and the elderly were displaced. This study was conducted in North Gondar, which is located 761 kilometres from Addis Ababa. There are three IDP sites (Dabat, Debark and Zarima) that house people who have experienced significant loss of property, fatal accidents and displacement. The first site was Dabat, with a total of 2084 people, of whom 874 are 18 and older. The second site was Debark, which holds 2430 people, 1320 of whom are 18 years and older. The third site was Zarima, with a total of 6470 people, of whom 3601 are 18 and older . The total population in all three sites is 10 984, with 5795 (3913 females and 1882 males) people aged 18 and above.

### Source and study population

Our source populations were all IDP aged 18 and above assigned to the temporary camps. The study population was IDP aged 18 and above who were available in temporary camps during the study period. IDP aged 18 and above who were available in the assigned camps at the time of the data collection period were included, whereas individuals who were unable to communicate and acutely sick at the time of the data collection period were excluded.

### Sample size determination and sampling technique

The sample size was calculated using the single population proportion formula by considering the following assumptions: the prevalence of suicidal ideation was estimated at 20.5% in a study conducted in Ethiopia among Eritrean refugees,^29^ with a 95% CI, a 3% margin of error and a 10% non-response rate. By applying the formula 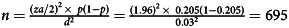 with a 10% non-response rate, the final sample size was 765. In the recruitment of participants, simple random sampling was used to gain a total sample of 765 participants. Because the sample was drawn from three different locations, and in order to ensure that the sample was representative of the population, proportional allocation was employed. The leaders of each site provided the lists of participants, who were then selected through a computer-generated random method.

### Data collection procedures and tools

The data collection supervisors were two mental health professionals, whereas the interviewers were six nursing professionals working in the internally displaced community camps employed by the United Nations Children's Emergency Fund (UNICEF) and the International Organization for Migrants (IOM). They were trained for two days on how to handle the study tools and terms regarding mental health, as well as important interviewing techniques. Before data collection, all participants were given detailed information about the study. Written and verbal consent was obtained from participants after explaining all the purposes, risks, benefits and confidentiality of the information, and the voluntary nature of participation. Only individuals who gave their permission were interviewed. Separate structured interviews were conducted with each participant, without any information contamination, to establish their safety and privacy.

The selected participants were invited to a temporary hall (a large tent at the centre) set up by IOM and UNICEF within the temporary camp. During the interviews, five participants (four females and one male) cried and could not finish the interview. A referral system was implemented for these participants to go to the nearest hospital for quick psychological therapy. Other participants with suicidal ideation and attempts were advised to consult healthcare providers and mental health professionals. Four participants (two females and two males) were not willing to finish the interview, giving time loss as the reason. The interview was conducted in Amharic, a native and familiar language to participants and interviewers. Both supervisors and data collectors were familiar with the community and its language, and sociocultural and environmental contexts.

Suicidal ideation and attempt were measured using ‘yes’ or ‘no’ questions, with 1 representing ‘yes’ and 0 representing ‘no’. The questions were adapted from the Composite International Diagnostic Interview (CIDI) module of the WHO's World Mental Health Survey Initiative.^30^ In Ethiopia, it has been validated and shown to be used in both community and clinical settings.^31^ If the respondents answered yes to the question ‘Have you seriously thought about death by suicide during the last 12 months?' this was defined as suicide ideation. A suicide attempt was defined if the respondent answered yes to the question ‘Have you attempted suicide during the last 12 months?’

A Patient Health Questionnaire - nine items (PHQ-9) was applied to assess depression, and each item had a four-point Likert scale ranging from 0 to 3 (0 = not at all to 3 = nearly every day). The score range was 0–27, and individuals who scored 10 and above were considered to have depression.^32^ The Generalised Anxiety Disorder - seven items (GAD-7) scale was applied to assess anxiety. It is a seven-item questionnaire to measure the level of anxiety among the respondents during the preceding two weeks. Every item has a score ranging from 0 (not at all) to 4 (nearly every day). The scores of all items were added to get the total scores, with a range of 0 to 21 and a minimum cut-off point of 10 or more suggesting that the person has anxiety;^33^ we also used the Post-traumatic Stress Disorder Checklist for the Diagnostic and Statistical Manual (DSM-5) (PCL-5 with extended criteria) to assess post-traumatic stress symptoms. A total score was computed by adding the 20 items; scores range from 0 to 80 with a five-point Likert scale(0 = ‘not at all’ to 4 = ‘extremely’) with a cut-off point of ≥33.^34^

The Alcohol, Smoking, and Substance Involvement Screening Test (ASSIST^35^) was applied to assess substance use. Current users of substances and those who had ever been users were defined as having used at least one of any specific substance for a non-medical purpose within the last three months (alcohol, khat, tobacco), and having used at least one of any specific substance for a non-medical purpose at least once in their lifetime (alcohol, khat, tobacco), respectively.^36^ We used the Oslo-3 Social Support Scale (OSS-3) to assess social support. OSS-3 has a total of 14 scores and is classified into three broad categories: poor support: 3–8, moderate support: 9–11 and strong support: 12–14.^37^

### Data quality control

The questionnaire was initially prepared in English and first translated into the local Amharic language for participants, then back-translated to English by an independent person to maintain consistency. A pre-test was done on 5% of the samples (n = 39) from the Kebero-Meda IDP site (in the Central Gondar zone) to test whether the questions were understandable to the pre-test sample, and necessary amendments were made. (Cronbach's alpha of CIDI = 0.86, PHQ-9 = 0.79, GAD-7 = 0.82, PCL-5 = 0.89 and OSS-3 = 0.86.) Supervisors provided oversight to data collectors throughout the data collection process. The principal investigator, supervisors and data collectors met on a regular basis to discuss and resolve any issues that occurred during data collection. Before data entry, the collected data were vetted and verified for accuracy.

### Data processing and analysis

The data were checked, coded and entered into Epi-Data version 4.2.0 for Windows and exported to STATA version 14 for Windows for analysis. The descriptive examination of participants was employed in response to the CIDI assessment tool to determine the prevalence of suicidality. We removed an extreme value of a continuous variable age, specifically, three male and one female participant. A bivariable logistic regression analysis was performed to find the association of sociodemographic, clinical, substance-related and psychosocial variables, to predict participant suicidal risk. Variables with a *P*-value of less than 0.2 in the bivariable logistic regression analysis were entered into the multivariable logistic regression model to identify significant suicide-related factors. Then multivariable logistic regression was computed, and variables with a *P*-value of ≤ 0.05 were considered statistically significant factors for suicide. The adjusted odd ratio (AOR) with a 95% CI was calculated. The Hosmer–Lemeshow test was applied to examine the goodness of the model's fitness (0.17 and 0.54) for suicidal ideation and attempt, respectively. The result shows that the model was fitted to measure the processed data by the logistic regression analysis.

### Declarations

#### Ethical approval and informed consent

The authors assert that all procedures contributing to this work comply with the ethical standards of the relevant national and institutional committees on human experimentation, and with the Helsinki Declaration of 1975 as revised in 2008**.** Ethical clearance was approved by the University of Gondar Ethical Review Board (ref no. SOM/1540/2022), and administrative approval was received from the North Gondar administrative office and the camp authorities before data collection. Before data collection, all participants were given detailed information about the study. After explaining all the purposes, risks, benefits and confidentiality of the information, and voluntary nature of the participation, both verbal informed consent for participants who could not read and write and written informed consent for participants who could read and write were obtained. Only individuals who gave their permission were interviewed. Five participants who had suffered from active suicidal thoughts were referred to the nearby hospital (Dabat Hospital). Other participants with suicidal ideation and attempts were advised to consult healthcare providers and mental health professionals.

## Results

### Sociodemographic characteristics of respondents

Sociodemographic factors include the gender, age, marital status, religion and educational status of respondents. A total of 751 participants out of 765 were included in the study, with a response rate of 98.2%. About two-thirds of respondents (504) (67.1%) were female. The mean age (SD) of the respondents was 34.34 (±10) years old, ranging from 18 to 58 years; 483 (64.3%) were in the age range 26–49 years; 433 (57.6%) were married; and 709 (94.4%) were Orthodox. In terms of educational level, 284 (37.8%) had no formal education, whereas 64 (8.5%) had college or higher education (Table 1).

**Table 1:** Sociodemographic characteristics of war-affected internally displaced people in northwest Ethiopia, 2022 (*n* = 751)

Variable	Category	Frequency	Percentage
Gender	Male	247	32.9
	Female	504	67.1
Age	18–25	179	23.8
	26–49	483	64.3
	>49	89	11.9
Marital status	Married	433	57.6
	Single	168	22.4
	Divorced	78	10.4
	Widowed	72	9.6
Religion	Orthodox	709	94.4
	Muslim	38	5.1
	Protestant	4	0.5
Educational status	No formal education	284	37.8
	Primary	252	33.6
	Secondary	151	20.1
	College and above	64	8.5

### Clinical characteristics of respondents

More than half of the participants, 403 (53.7%), scored ≥33 based on PCL-5, and 388 (51.7%) scored ≥10 based on PHQ-9. Almost half of the respondents, (369, 49.1%), scored ≥10 using GAD-7, 65 (8.7%) had a chronic medical illness and 80 (10.6%) had family with a history of mental illness (Table 2).

**Table 2:** Description of clinical characteristics of war-affected internally displaced people in northwest Ethiopia, 2022 (*n* = 751)

Variable	Category	Frequency (*n* = 751)	Percentage
Depression	Yes	388	51.7
	No	363	48.3
Anxiety	Yes	369	49.1
	No	382	50.9
Post-traumatic stress symptoms	Yes	403	53.7
	No	348	46.3
Chronic medical illness	Yes	65	8.7
	No	686	91.3
Family history of mental illness	Yes	80	10.6
	No	671	89.4

### Substance use and psychosocial characteristics of respondents

Concerning substance use, more than half of the respondents (426, 56.7%) had used alcohol, and 238 (31.7%) were current alcohol users. A total of 16 (2.1%) of respondents had smoked cigarettes during their lives, whereas 6 (0.8%) of them were current smokers. In all, 46 (6%) of respondents had used khat at some point in their lives, and 28 (3.7%) were current khat users. Concerning the psychosocial characteristics of respondents, 84 (11.9%) had lost a family member or loved one, whereas 347 (47.3%), 285 (38%) and 110 (14.6%) had poor, moderate and strong social support, respectively (Table 3).

**Table 3:** Description of substance use and psychosocial characteristics of war-affected internally displaced people in northwest Ethiopia, 2022 (*n* = 751)

Variable	Category	Frequency (*n* = 751)	Percentage
Use of alcohol ever	Yes	426	56.7
	No	325	43.3
Use of khat ever	Yes	46	6.1
	No	705	93.9
Use of tobacco ever	Yes	16	2.1
	No	735	97.9
Current use of alcohol	Yes	238	31.7
	No	513	68.3
Current use of khat	Yes	28	3.7
	No	723	96.3
Current use of tobacco	Yes	6	0.8
	No	745	99.2
Death of family member	Yes	84	11.2
	No	667	88.8
Social support	Poor	356	47.4
	Moderate	285	38.0
	Strong	110	14.6

### Prevalence of suicide ideation and attempts among war-affected IDP

The 12-month prevalence of suicidal ideation and attempt among respondents was found to be 22.4% (95% CI: 19.5%, 25.4%) and 6.7% (95% CI: 5.1%, 8.7%), respectively, whereas the gender-specific distribution was 26.58%, 13.76 for suicidal ideation and 8.53 and 2.83% for suicidal attempt among females and males, respectively. Among respondents who had attempted suicide, 37 (74%) had attempted it once, 11 (22%) had attempted it twice and 2 (4%) of them had attempted it more than twice. Nearly two-thirds (64%) used poisoning, and four (8%) used sharp tools.

About 17 (34%) of them claimed to have done so as a cry for help (seeking attention), and 16 (32%) claimed to have made a serious attempt but were only saved by luck. Another 10 (20%) claimed to have failed because of the method they used, and others claimed to have been interrupted by family members, family-related thoughts and religious concerns. They attempted it for a variety of reasons, including 22 (44%) because of poverty or financial loss, 12 (24%) because of family conflict, 11 (22%) because of the death of a family member or significant loved one, and 5 (10%) because of mental illness.

### Factors associated with suicidal ideation among war-affected IDP

Female gender, age (18–25 years and >49), marital status (single, divorced and widowed), having depression, anxiety, post-traumatic stress symptoms, family with a history of mental illness, current khat use and poor social support were factors associated with suicidal ideation at a *P*-value ≤ 0.2. The multivariable analysis identified that female gender, having depression or post-traumatic stress symptoms, family with a history of mental illness and poor social support were factors significantly associated with suicidal ideation at a *P*-value of ≤ 0.05. Females had more than twice the odds of suicidal ideation compared with males (AOR = 2.54, 95% CI: 1.62, 4.00). Those who had depression had approximately four (AOR = 3.57, 95% CI: 2.32, 5.49) times higher odds of suicidal ideation than those who did not have depression.

Respondents who had post-traumatic stress symptoms had approximately three (AOR = 2.87, 95% CI: 1.88, 4.37) times higher odds of suicidal ideation than those who did not have post-traumatic stress symptoms. The odds of suicidal ideation were more than two (AOR = 2.42, 95% CI: 1.39, 4.21) times higher among those who had a family history of mental illness than among those without a family history of mental illness. Respondents with low social support had 2.29 (95% CI: 1.21–4.34) higher odds of suicidal ideation than those with high social support (Table 4).

**Table 4:** Bivariate and multivariate logistic regression analysis of variables and suicidal ideation among war-affected internally displaced people in northwest Ethiopia, 2022 (*n* = 751)

Explanatory variable	Category	Suicidal ideation		COR (95% CI)	AOR (95% CI)
		Yes	No		
Gender	Male	34	213	1.00	1.00
	Female	134	370	2.27 (1.50, 3.43)	2.54 (1.62, 4.00)***
Age	18–25	49	130	1.58 (1.06, 2.35)	1.25 (0.74, 2.12)
	26–49	93	390	1.00	1.00
	>49	26	63	1.73 (1.04, 2.88)	1.27 (0.69, 2.34)
Marital status	Married	77	356	1.00	1.00
	Single	45	123	1.69 (1.11, 2.58)	1.47 (0.84, 2.55)
	Divorced	22	56	1.82 (1.05, 3.15)	1.33 (0.72, 2.45)
	Widowed	24	48	2.31 (1.34, 4.00)	1.37 (0.72, 2.61)
Depression	No	39	324	1.00	1.00
	Yes	129	259	4.14 (2.79, 6.13)	3.57 (2.32, 5.49)***
Anxiety	No	57	315	1.00	1.00
	Yes	101	268	1.77 (1.25, 2.51)	1.07 (0.72, 1.60)
PTSS	No	42	306	1.00	1.00
	Yes	126	277	3.31 (2.25, 4.87)	2.87 (1.88, 4.37)***
Family history of mental illness	No	538	133	1.00	1.00
	Yes	35	45	3.15 (1.95, 5.08)	2.42 (1.39, 4.21)**
Current khat use	No	158	565	1.00	1.00
	Yes	10	18	1.98 (0.89, 4.39)	2.07 (0.83, 5.14)
Social support	Strong	15	95	1.00	1.00
	Moderate	40	245	1.03 (0.55, 1.96)	1.19 (0.59, 2.35)
	Poor	113	243	2.95 (1.64, 5.31)	2.29 (1.21, 4.34)*

*** = *P* value < 0.001, ** = *P* value < 0.01, * = *P* value < 0.05, Hosmer–Lemeshew = 0.17.

PTSS, post-traumatic stress symptoms; COR, crude odds ratio; AOR, adjusted odds ratio.

### Factors associated with suicidal attempts among war-affected IDP

Female gender, age (18–25 years and >49), marital status (single, divorced and widowed), death of family members, having depression, anxiety, post-traumatic stress symptoms, chronic medical illness, family with a history of mental illness, current khat use and poor social support were factors significantly associated with suicidal ideation at a *P*-value ≤ 0.2. In multivariable analysis, female gender, death of a family member, depression and family with a history of mental illness were factors significantly associated with suicidal attempts at a *P*-value of ≤ 0.05. Females were three times more likely than males to attempt suicide (AOR = 3.39, 95% CI: 1.44, 8.00).

Displaced people who had lost a family member or significant loved one were three times more likely to attempt suicide (AOR = 3.10, 95% CI: 1.45, 6.65) than those who had not lost a family member. Respondents with depression were nearly four times more likely to attempt suicide (OR = 4.69, 95% CI: 1.76, 11.08), and those with a family history of mental illness were nearly four times (AOR = 3.73, 95% CI: 1.76, 7.88) more likely (Table 5).

**Table 5:** Bivariate and multivariate logistic regression analysis of variables and suicidal attempts among war-affected internally displaced people in northwest Ethiopia, 2022 (*n* = 751)

Variable	Category	Suicidal attempt		COR (CI)	AOR (CI)
		Yes	No		
Gender	Male	7	240	1.00	1.00
	Female	43	461	3.20 (1.42, 7.22)	3.39 (1.44, 8.00)**
Age	18–25	21	158	2.92 (1.55, 5.49)	2.08 (0.91, 4.80)
	26–49	21	462	1.00	1.00
	>49	8	81	2.17 (0.93, 5.07)	1.33 (0.48, 3.67)
Marital status	Married	17	416	1.00	1.00
	Single	20	148	3.31 (1.69, 6.48)	1.96 (0.82, 4.69)
	Divorced	7	71	2.41 (0.96, 6.03)	2.01 (0.74, 5.44)
	Widowed	6	66	2.22 (0.85, 5.85)	1.34 (0.46, 3.91)
Death of family member	No	37	630	1.00	1.00
	Yes	13	71	3.12 (1.58, 6.14)	3.10 (1.45, 6.65)**
Depression	No	7	356	1.00	1.00
	Yes	43	345	6.34 (2.81, 14.28)	4.69 (1.99, 11.08)***
Anxiety	No	17	365	1.00	1.00
	Yes	33	336	2.11 (1.15, 3.85)	1.34 (0.68, 2.65)
PTSS	No	18	330	1.00	1.00
	Yes	32	371	1.58 (0.87, 2.87)	1.17 (0.59, 2.29)
Chronic medical illness	No	43	643	1.00	1.00
	Yes	7	58	1.80 (0.78, 4.19)	1.56 (0.57, 4.23)
Family history of mental illness	No	33	638	1.00	1.00
	Yes	17	63	5.22 (2.75, 9.89)	3.73 (1.76, 7.88)**
Current khat use	No	46	677	1.00	1.00
	Yes	4	24	2.45 (0.82, 7.37)	2.13 (0.59, 7.75)
Social support	Strong	4	106	1.00	1.00
	Moderate	12	273	1.16 (0.37, 3.69)	1.46 (0.43, 4.95)
	Poor	34	322	2.79 (0.97, 8.07)	1.95 (0.62, 6.08)

COR, crude odds ratio; AOR, adjusted odds ratio; PTSS, post-traumatic stress symptoms.

*** = *P* value < 0.001, ** = *P* value < 0.01, Hosmer–Lemeshew = 0.54.

## Discussion

This study was one of the first to determine the prevalence and associated factors of suicidal ideation and attempts among IDP in Ethiopia. While this makes it challenging to put the findings in the context of past studies, it also offers a chance to evaluate the application of findings from other settings and to motivate more research with vulnerable and understudied people in analogous circumstances. In general, we found that these people had a high incidence of suicide ideation, with about one out of every five respondents reporting having had such thoughts. These results have to be concerning and are undoubtedly in the study's favour, and it is crucial to raise awareness of the pain and mental risks among displaced people. This study may be one of the reliable pieces of information that specific stakeholders use to execute and carry out an action. To lower the danger of fatalities, the industry norm for psychoactive drug trials has been to screen out volunteers for suicidality. Unfortunately, many mental illnesses and psychosocial variables have a medically expected result of suicide.^38^

The 12-month prevalence of suicidal ideation among respondents was found to be 22.4% (95% CI: 19.5%, 25.4%). This finding was in line with studies carried out in Ethiopia among Eritrean refugees,^29^ in Mississippi^16^ and Colombia.^15^ However, it was higher than studies carried out on conflict affected people in Ireland^39^, and Burundian refugees.^10^ Based on our findings, these study populations also have definite suicide thoughts at rates that are higher than those seen in community samples of adults in LMICs over the past year.^40^ They are also in the middle of the range of past-month rates reported by refugees in camps in prior studies. Burundian refugees were familiar with the environment and lifestyle, which was better than recent migrants in this study population. New situations and recent crises might produce higher stress levels, more challenged lifestyles, and more sociocultural and economic hardships than crises with a longer duration.^41^ On the other hand, women made up two-thirds of the respondents in this study, although men and women in the Ireland study^39^ were about equally represented, so we can conclude that the rate of suicide in this study is higher than the Ireland study because women were more likely than males to report suicidal ideation. Additionally the sample size in the study conducted in Ireland is higher than the currentdy study, and the sampling technique in prior study is multi-stage cluster sampling technique, while in the current study the technique was simple random sampling technique.^42^ However, it was lower than findings conducted in Tanzania among Burundian refugees^10^ and in Nigeria.^18^ In this study, the population of internally displaced individuals had a high social bond, network, integration, group relations and strong cultural background. These aspects might help people cope better with life's stresses, reduce the incidence of depression and substance misuse, facilitate recovery, enhance social support and provide sources of hope and meaning.^43^ Since most of the respondents (94.4%) were followers of the Orthodox religion, those who die by suicide cannot be absolved, and dying by suicide is completely forbidden by the Orthodox doctrine. This might reduce the incidence of suicide in this population.

In this study, the 12-month prevalence of suicidal attempts was found to be 6.7% (95% CI: 5.1%, 8.7%). This finding is in line with other studies carried out in Ethiopia,^44^ but it was higher than other studies carried out in LMICs.^16,19^ The discrepancy might be there because almost two-thirds of the respondents were females, who are more likely to attempt suicide than males.^45^ However, the magnitude was lower than studies conducted among Colombian displaced people.^15^ In Colombia, respondents were adolescents in the age range of 12–17, who were more likely to have attempted suicide than adults.^46^ They also faced violence, direct exposure to war and traumatic life events, stressors and higher rates of psychopathology than this study population. This might cause an increase in susceptibility to suicide.

The odds of having suicidal ideation and attempts among female respondents were about 2.54 and 3.39 times higher than those of male respondents, respectively. This is in line with studies conducted in Uganda.^47^ In this study, females experienced more presentations with post-traumatic stress symptoms and depressive symptoms, which can expose them to suicidal ideation and attempts. It might be because higher psychosocial stressors and more depressive symptoms and childhood maltreatment histories are experienced by women. Females are more biologically vulnerable, highly sensitive to stressful events and have more maladaptive defence mechanisms than males.^48^ According to prior studies, women's gender is a risk factor for suicide thoughts and conduct among population samples in LMICs, and the higher incidence of the various kinds of suicidality among women compared with males is consistent with those findings.^49^

Participants who had depression were 3.5 and 4.7 times more likely to have suicidal ideation and attempts than their counterparts without depression, which was supported in previous studies.^50^ It is possible that a depressed individual frequently thinks about entirely different things before and after a depressed episode. This may be the outcome of a neurochemical imbalance that causes the person to be unaware of the resources available to alleviate the pain they are experiencing.^51^

Respondents who had a family history of mental illness were 2.4 and 3.7 times more likely to have suicidal ideation and attempts than respondents without a family history of mental illness, respectively. This finding is consistent with other studies carried out in Ethiopia.^29,44^ In the familial clustering of suicide in families with mental illness, there may be genetic factors for those individuals to develop a mental illness. Families with known mental illnesses might be genetically prone to suicide ideation and attempts,^52^ and the offspring might be exposed to suicidal ideation and attempts.^53^

The odds of having suicidal ideation were 2.8 times higher among respondents who had post-traumatic stress symptoms than their counterparts. This is consistent with other studies carried out in Ethiopia.^29^ Those who had post-traumatic stress symptoms experienced distressing traumatic memories, anger and poor control of impulses. A person who is exposed to reminders and flashbacks related to anxiety and fear may feel that those are too much to overcome, which can trigger suicidal ideation.^25^

Individuals who had poor social support had more than two times higher odds of suicide ideation than those who had strong social support. This is consistent with a study carried out among Burundian refugees in Tanzania.^10^ Those who had poor social support were not expressing their stressful experiences, lack of confidence, low self-esteem, and negative emotional and psychological conditions.^54^ It suggests that one of the most important resources in the camps is the ability to obtain financial assistance from others in times of need. It is interesting to note that a lower income was associated with a higher risk of exhibiting suicide. Given the small numbers of participants in the higher income and suicide risk groups, it is difficult to interpret this finding, but it may suggest that, in a situation where poverty is pervasive, for example, having better economic opportunities than the majority could also be problematic because it encourages discrimination and envy among community members. Respondents who lost family members or significant loved ones had three times higher odds of attempting suicide. The later effect of a loss event may be a strong precipitator of mental illness. Those who had lost their families may have lost hope as well as significant support.^55^

### Limitations of the study

It is important to take into account the limitations of this study. The study's cross-sectional design did not draw conclusions about the causes and effects of each factor. A proper assessment of past medical and mental status was not possible either. Because the study measures began within the last 12 months, there may be social desirability bias and recall bias in remembering past events. The other limitation of this study is that the authors did not validate the inter-rator reliability of data collectors.

## Conclusions and recommendations

This study's findings concluded that more than one in five and one in twenty internally displaced people suffered suicidal ideation and suicidal attempts, respectively. Suicidal ideation was significantly associated with gender (female respondents), depression, post-traumatic stress symptoms, a family history of mental illness and a lack of social support. In addition, there was a substantial association found between suicidal attempts and gender (females), depression, a family history of mental illness and the death of a family member or significant others. This implies that special considerations and urgent interventions must be applied for females, those who have depression or post-traumatic stress symptoms, a family history of mental illness, poor social support and the loss of family members. Since the authors in this study did not validate the inter-rator reliability, we recommend that future researchers conduct inter-rator reliability and do a longitudinal study to clearly allow for inferences of causal relationships among variables. The specific significance of issues like insecure housing, lack of food, suffering violence directly or witnessing conflict, family or community displacement and family separation are not assessed in this study. Consequently, it is recommended that future researchers develop and utilise questions that are pertinent to people residing in LMICs, particularly those that reflect the actual circumstances of IDP.

## Data Availability

On reasonable request, the corresponding author will provide the data-set that was employed and/or analysed for the current study.
